# Genomics of the expanding pine pathogen *Lecanosticta acicola* reveals patterns of ongoing genetic admixture

**DOI:** 10.1128/msystems.00928-23

**Published:** 2024-02-16

**Authors:** Marina Marcet-Houben, Fernando Cruz, Jéssica Gómez-Garrido, Tyler S. Alioto, Juan Carlos Nunez-Rodriguez, Nebai Mesanza, Marta Gut, Eugenia Iturritxa, Toni Gabaldon

**Affiliations:** 1 Barcelona Supercomputing Centre (BSC-CNS), Barcelona, Spain; 2 Institute for Research in Biomedicine (IRB Barcelona), The Barcelona Institute of Science and Technology, Barcelona, Spain; 3 Centro de Investigación Biomédica En Red de Enfermedades Infecciosas (CIBERINFEC), Barcelona, Spain; 4 CNAG-CRG, Centre for Genomic Regulation (CRG), Barcelona Institute of Science and Technology (BIST), Barcelona, Spain; 5 Universitat Pompeu Fabra (UPF), Barcelona, Spain; 6 Instituto Vasco de Investigación y Desarrollo Agrario (BRTA), Arkaute, Araba, Spain; 7 Catalan Institution for Research and Advanced Studies (ICREA), Barcelona, Spain; State Key Laboratory of Mycology, Institute of Microbiology, Chinese Academy of Sciences, China

**Keywords:** comparative genomics, plant pathogen, population genomics, admixture, *Lecanosticta acicola*, needle blight

## Abstract

*Lecanosticta acicola* is the causal agent for brown spot needle blight that affects pine trees across the northern hemisphere. Based on marker genes and microsatellite data, two distinct lineages have been identified that were introduced into Europe on two separate occasions. Despite their overall distinct geographic distribution, they have been found to coexist in regions of northern Spain and France. Here, we present the first genome-wide study of *Lecanosticta acicola*, including assembly of the reference genome and a population genomics analysis of 70 natural isolates from northern Spain. We show that most of the isolates belong to the southern lineage but show signs of introgression with northern lineage isolates, indicating mating between the two lineages. We also identify phenotypic differences between the two lineages based on the activity profiles of 20 enzymes, with introgressed strains being more phenotypically similar to members of the southern lineage. In conclusion, we show undergoing genetic admixture between the two main lineages of *L. acicola* in a region of recent expansion.

**IMPORTANCE:**

*Lecanosticta acicola* is a fungal pathogen causing severe defoliation, growth reduction, and even death in more than 70 conifer species. Despite the increasing incidence of this species, little is known about its population dynamics. Two divergent lineages have been described that have now been found together in regions of France and Spain, but it is unknown how these mixed populations evolve. Here we present the first reference genome for this important plant pathogenic fungi and use it to study the population genomics of 70 isolates from an affected forest in the north of Spain. We find signs of introgression between the two main lineages, indicating that active mating is occurring in this region which could propitiate the appearance of novel traits in this species. We also study the phenotypic differences across this population based on enzymatic activities on 20 compounds.

## INTRODUCTION

*Lecanosticta acicola* is a plant pathogenic fungus causing brown spot needle blight in at least 30 different species of pine trees (1, 2). Currently, 70 conifer taxa are determined hosts of this pathogen, including hybrids or subspecies of known susceptible species (3). This disease causes severe defoliation that results in a significant reduction in growth when more than 25% of the needles are affected (4) and, in extreme cases, causes tree mortality (2, 5). *L. acicola* has a broad distribution in northern hemisphere regions across America, Europe, and Asia (6), with recent studies reporting geographic expansions and an increased range of host species (1, 7). Nowadays, the presence of *L. acicola* is confirmed in 24 of the 27 European countries (3). Severe defoliation events and outbreaks have escalated worldwide reducing forest productivity and increasing tree mortality (3, 4, 8, 9) and have been associated with climatic changes, underscoring the importance of studying this fungus in the current context of global warming (9).

*L. acicola* is a heterothallic species with two different idiomorphs named MAT 1-1 and MAT 1-2. It can reproduce clonally *via* asexual conidia that are dispersed predominantly by rain splash and dew, accounting for localized infections. Sexual ascospores are dispersed through air and facilitate longer-range dispersal (1, 10), which, in turn, increases the severity of the disease (4). The presence of the two mating types in similar frequencies in the same area suggests active sexual reproduction (1). A previous report showed the presence of both idiomorphs in the Basque Country (northern Spain) (4). Active sexual reproduction was later confirmed by the presence of the sexual ideomorph of *L. acicola* (*Mycosphaerella dearnessii*) in *Pinus radiata* needles collected in the area of study. This was confirmed by both morphological and molecular methods (11).

The genus *Lecanosticta* comprises nine described species, of which *L. acicola* is so far the only one found outside of Mexico and Central America (12), where this species is thought to have originated (13). Janousek et al. (1) established that an unknown ancestral *L. acicola* population gave rise to a population in North America which then split into a second American population located more toward the south of the USA. These two lineages were dubbed as the northern lineage (NL) and the southern lineage (SL) and from there, the species expanded to different parts of the world. It is thought that *L. acicola* entered Europe twice with the NL expanding through central and eastern Europe and the SL expanding through southern Europe, which reflects the actual distribution of the two lineages in Europe.

Using genotyping of marker genes, van der Nest et al. (12) identified three different lineages of *L. acicola*, one located uniquely in Mexico while the other two were related to the NL and SL described by Janoušek et al. (1). In a more recent study (6) based on microsatellite sequences from a set of 650 strains from 27 different countries, both the SL and NL were found to be structured in subpopulations that were mostly congruent with geographic location. For instance, SL isolates from France, Spain, and Portugal were forming a different subpopulation than SL isolates from southern-eastern states in the USA. Notably, the study found, for the first time, the two lineages coexisting in the same geographic region (France). Based on the patterns found, the authors proposed that the South European strains were the result of an admixture event between the two lineages.

Due to the absence of a reference genome for *L. acicola,* all studies so far have been based on marker genes or microsatellites, providing limited resolution. Similarly, our knowledge about genetic and phenotypic differences between the two lineages is poor, although a reduced ability to sporulate at higher temperatures (28°C to 32°C) in the NL suggests phenotypic differentiation (14).

A recent population study in the Basque Country region revealed the predominance of the SL even when strains from the NL were also detected. There are different hypotheses as to why the SL isolates are more widespread in this region. On the one hand, it has been suggested that they were introduced first, which is corroborated by the fact that the NL isolates are only found in the newly established arboretum. On the other hand, the dominance of the SL might be connected with differences in virulence between SL and NL isolates. SL isolates have been reported to be more virulent to *Pinus spp*. than NL ones except for *P. sylvestris* and this condition may lead to this imbalance between lineages.

The impact of *L. acicola* disease is determined by the presence of susceptible hosts and their abundance and also by the pathogen genetic diversity found in a region (3). Knowledge of genetic diversity and mating capacity of pathogens is sometimes limited by the tools used for its determination which could influence the effectiveness of the management measures established to control a disease.

Here, we address the important gap of missing genomic information for this key plant pathogen by assembling and annotating the first reference genome of *L. acicola* and showcasing its potential by performing a comprehensive population genomic analysis of 69 additional isolates from northern Spain.

## RESULTS AND DISCUSSION

### Genome assembly and phylogenomics

**Fig 1:**
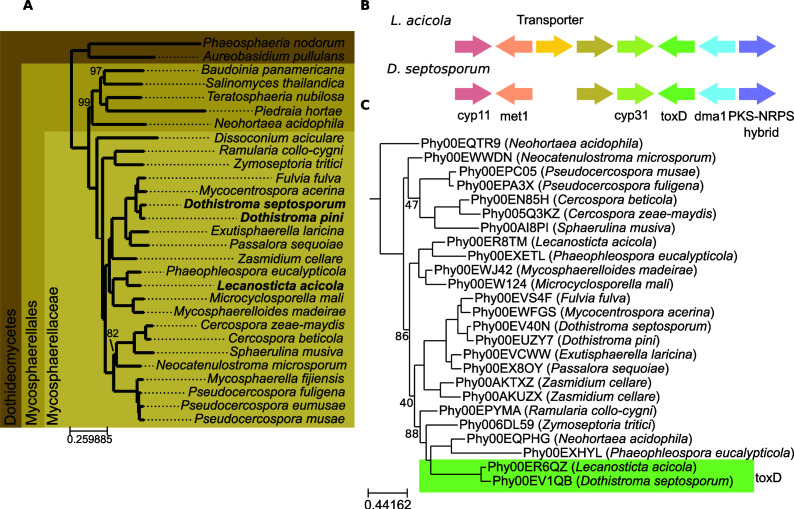
(A)- Phylogenetic tree representing the evolutionary relationships among the species included in the phylome. The tree is based on a maximum likelihood analysis of a concatenated alignment of 1,092 genes (see Methods), trimmed to delete columns with more than 20% gaps. The phylogenetic tree was reconstructed using IQTREE using the LG + F + R8 model according to the BIC criterion. Rapid bootstrap was calculated and nodes with less than 100% support are indicated. Species indicated in bold are pathogens of pine trees. (B)- Representation of the gene cluster found shared between *L. acicola* and D. *septosporum*. (C)- Gene tree representing the evolution of ToxD as obtained from the phylome reconstruction. Bootstrap values below 90 are marked on the tree.

The reference genome of *L. acicola* AI6289 was built using a combination of long Oxford Nanopore and short Illumina reads (see Materials and Methods). This resulted in a genome assembly of 30.4 Mb with an N50 of 290,931 bp. The gene completeness reported by BUSCO v5.4.7 is 99.7%. The gene annotation pipeline produced 10,195 protein-coding genes (see Materials and Methods). We compared this proteome with 28 other proteomes from sequenced species in the Dothideomycetidae lineage by reconstructing the *L. acicola* phylome, the complete collection of phylogenetic trees for each gene in the genome, which was used to derive orthology and paralogy relationships (see Materials and Methods and Table S1). The genome of *L. acicola* has been deposited in ENA (European Nucleotide Archive) (PRJEB62799) and the phylome data can be accessed in phylomeDB (https://phylomedb.org) with phylomeID 117 (15). These data represent the first genome-wide genetic and evolutionary resources for this important plant pathogen. Based on an alignment of conserved single-copy orthologs derived from the phylome, we inferred the evolutionary relationships across the considered Dothideomycetidae species (Fig. 1A). According to this phylogeny, the closest sequenced relative of *Lecanosticta* is *Phaeophleospora eucalypticola*. Other pine pathogens included in the phylome and often found in the same environment as *L. acicola* are *Dothistroma pini* and *Dothistroma septosporum* which were more distantly related. According to phylome analyses, *L. acicola* has 413 orphan genes, 6.1% of the proteome is predicted to be secreted and 3.2% encode CAZY proteins, these two values are close to the average in the whole species set (6.1% and 3.1%, respectively) (see Table S1). We searched for genes that could be specifically related to pine virulence by identifying trees where *L. acicola* and *Dothistroma* genes were monophyletic. This search identified seven neighboring genes LECACI7A004195P1 to LECACI7A004202P1, each being orthologous to *D. septosporum* genes that were also clustered. In *D. septosporum* this gene cluster was identified as the Hps1-Dma1 cluster, homologous to the cyclopiazonic acid gene cluster in *Aspergillus* (16). In that study, the production of this metabolite was not observed, leading the authors to conclude that either the cluster was producing a different compound, the proper condition for its expression had not been found, or it was a non-functional cluster. The two clusters maintain a perfectly conserved gene order with only an additional gene encoding a transporter found in *L. acicola* (see Fig. 1B). The use of evolclust (17) confirmed that the cluster was more conserved than expected, given the base pattern of gene order conservation between *L. acicola* and D. septosporum, and in addition, it indicated the cluster was exclusively conserved in these two species. The average protein sequence identity between orthologous genes in the cluster is 84%, which is much higher than the average identity among one-to-one orthologs between these two species (55.6% ± 21%). The gene tree topology (see Fig. 1C), the conserved gene order exclusive to the two species, and the atypical high similarity between the clustered genes in the two species, could point to horizontal transmission of the gene cluster between the two species as the alternative scenarios seem less parsimonious. The vertical inheritance of the cluster would imply that the common ancestor of *L. acicola* and *D. septosporum* had the cluster and that then it was lost on six different occasions with few genes remaining in the other species. Convergent evolution is also unlikely given that these genes are located in the same order and orientation and that orthologs present in other species do not appear to group with *L. acicola* and *D. septosporum* in the gene trees as would be expected. The possibility that this cluster is involved in the pathogenesis of pine trees, a shared trait of both species, is intriguing but requires additional research.

### Marker gene analysis of an expanding *L. acicola* population

**Fig 2:**
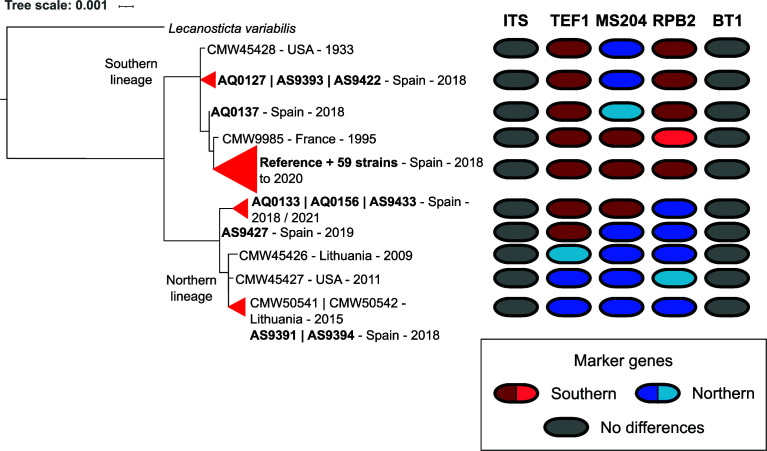
Phylogenetic tree based on the concatenation of marker genes. Strains sharing identical marker sequences were collapsed (indicated by triangles). In bold are strains sequenced in this study. On the right of the tree marker sequences are represented: each column represents a marker gene. Different marker haplotypes of the NL or SL are shown in shades of blue and red, respectively. Different shades of blue or red indicate strains that share the same pattern of SNPs for a given marker gene. Invariable markers are depicted in gray.

To gain insights into recent local expansions of spot needle blight in pine forests of the Basque region in Spain, we sequenced an additional set of 69 *L*. *acicola* isolates from this region (see Materials and Methods and Table S2). We then used PerSVade v1.2.04 (18) to calculate single nucleotide polymorphisms (SNPs) between each strain and the reference, which were used in subsequent analyses. To place our collection in the context of previous studies, we extracted the five marker genes used previously by van der Nest et al. (12): Nuclear ribosomal internal transcribed spacer (ITS), elongation factor 1-α gene region (TEF1), beta-tubulin-1 gene region (BT1), RNA polymerase II second largest subunit (RPB2), and the guanine nucleotide-binding protein subunit beta (MS204). Phylogenetic reconstruction of a concatenated alignment of the five markers in our 70 isolates (including the reference) and six from previous studies showed that most Basque isolates (64) grouped with isolates from the SL, as expected (Fig. 2). Out of those isolates, 60 strains, including the reference, had marker gene sequences identical to the other members of the SL provided by van der Nest et al. (12). Surprisingly, the remaining six isolates grouped closer to NL, with AS9391 and AS9394 having identical sequences to NL isolates from Lithuania (CMW50541 and CMW50542).

Remarkably, SNP patterns for the different markers suggest recombination among sequenced strains in the Basque Country (see Fig. 2). For instance, strains AQ0127, AS9393, and AS9422 while grouping closer to the SL contained the marker MS204 with a SNP pattern shared with the strains from the NL. On the other hand, AQ0133, AS9433, and AQ0156 grouped closer to strains from the NL but only shared the SNP patterns found in RPB2 while TEF1 and MS204 had the same SNP patterns as those found in the SL.

We then obtained the sequences for the 10 microsatellites defined by Janoušek et al. (19). These microsatellites have been used repeatedly to classify *L. acicola* strains in different haplotypes using capillary electrophoresis. When extracting the sequences from the reference genome, we noticed that MD1 and MD11 share the same genomic location, being MD11 a subsection of MD1, and that MD6 and MD10 are contiguous and overlap in a small region of 66 nucleotides. We defined new strain haplotypes according to our SNP patterns in the coordinates of the microsatellite regions. Based on this, we distinguish 39 different haplotypes, which provide increased resolution over the 21 microsatellite patterns observed with more traditional methods. We derived a microsatellite-based tree using the SNPs in these regions and compared it to a whole-genome-based tree reconstructed by concatenating all positions in the reference genome that had an SNP in at least one of the strains (see Materials and Methods). The topologies of the genome-based and microsatellite-based trees are largely different (see Fig. S1). This indicates that these microsatellite regions do not accurately capture the evolution of *L. acicola*, and suggest they are, similarly to the marker genes discussed above, subject to introgression events.

On the whole, these results underscore the importance of sequencing methodologies being introduced in the study and classification of *L. acicola,* as currently used marker genes and microsatellite regions lack sufficient resolution and can provide misleading results with respect to the evolution of the analyzed strains. Moreover, the presence of the two NL strains and the signs of recombination found in the marker genes show that *L. acicola* can reproduce sexually, as previously described (11), and that strains of the two lineages are actively mating in the studied region.

### Genome-wide analyses reveal introgression patterns among *L. acicola* isolates

**Fig 3:**
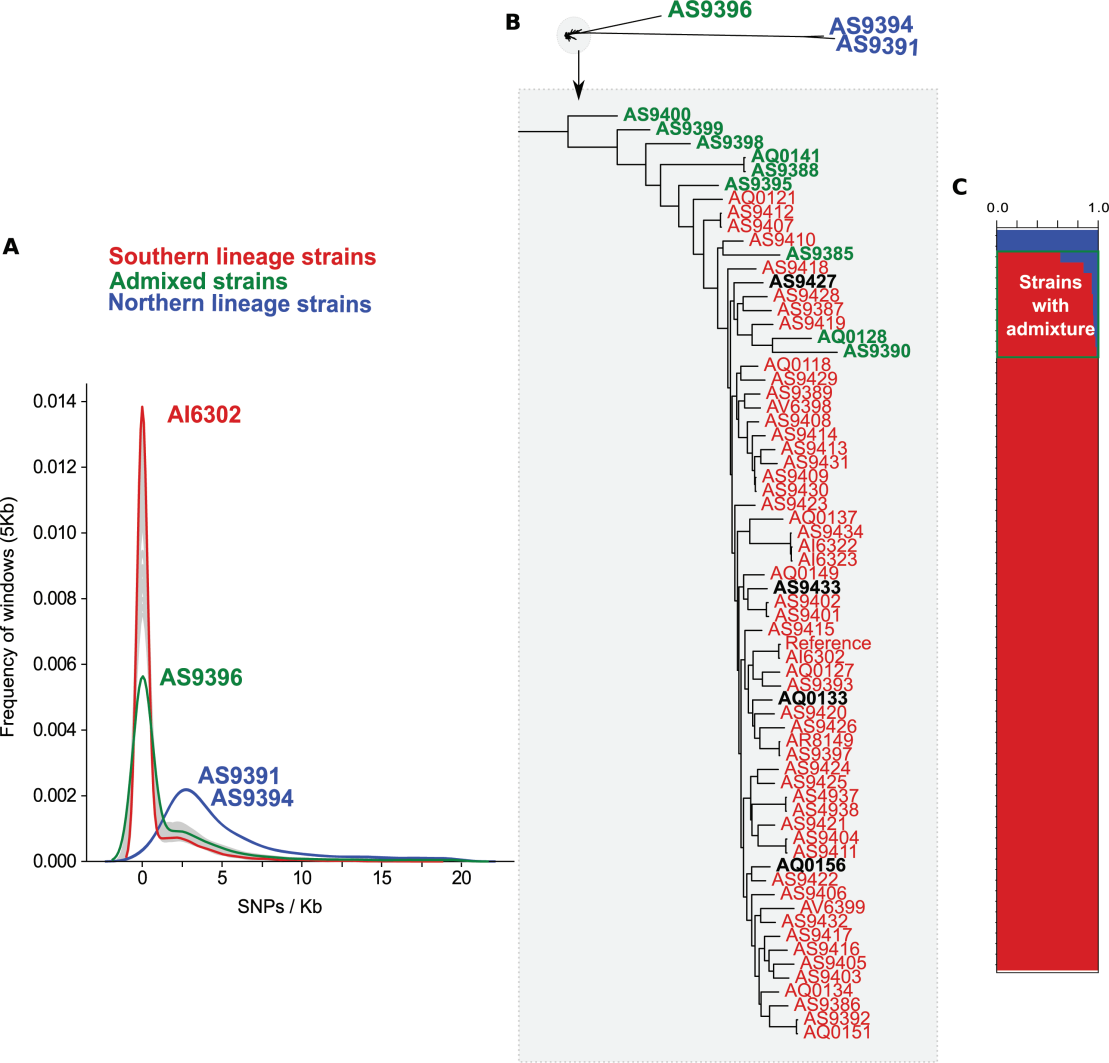
(A)- Frequency graph depicting the distribution of SNPs/Kb for each strain for windows of 5 Kb of the contigs longer than 100,000 base pairs. Green is the distribution belonging to the strain with an overall SNPs/Kb of 5. Blue is the distribution of the two NL strains. Red is an example of a SL strain (B)- On top is an unrooted tree showing the evolution of the 70 strains used in this study. Below it is a close-up image from the part of the phylogenetic tree holding strains belonging to the SL. Red are the strains considered purely from the SL. Green are the strains detected as admixed by Structure. Black are the strains that would have been classified as being from the NL based on marker genes. (C) Structure graph showing the two populations in blue and red. The green square indicates admixed isolates.

**Fig 4:**
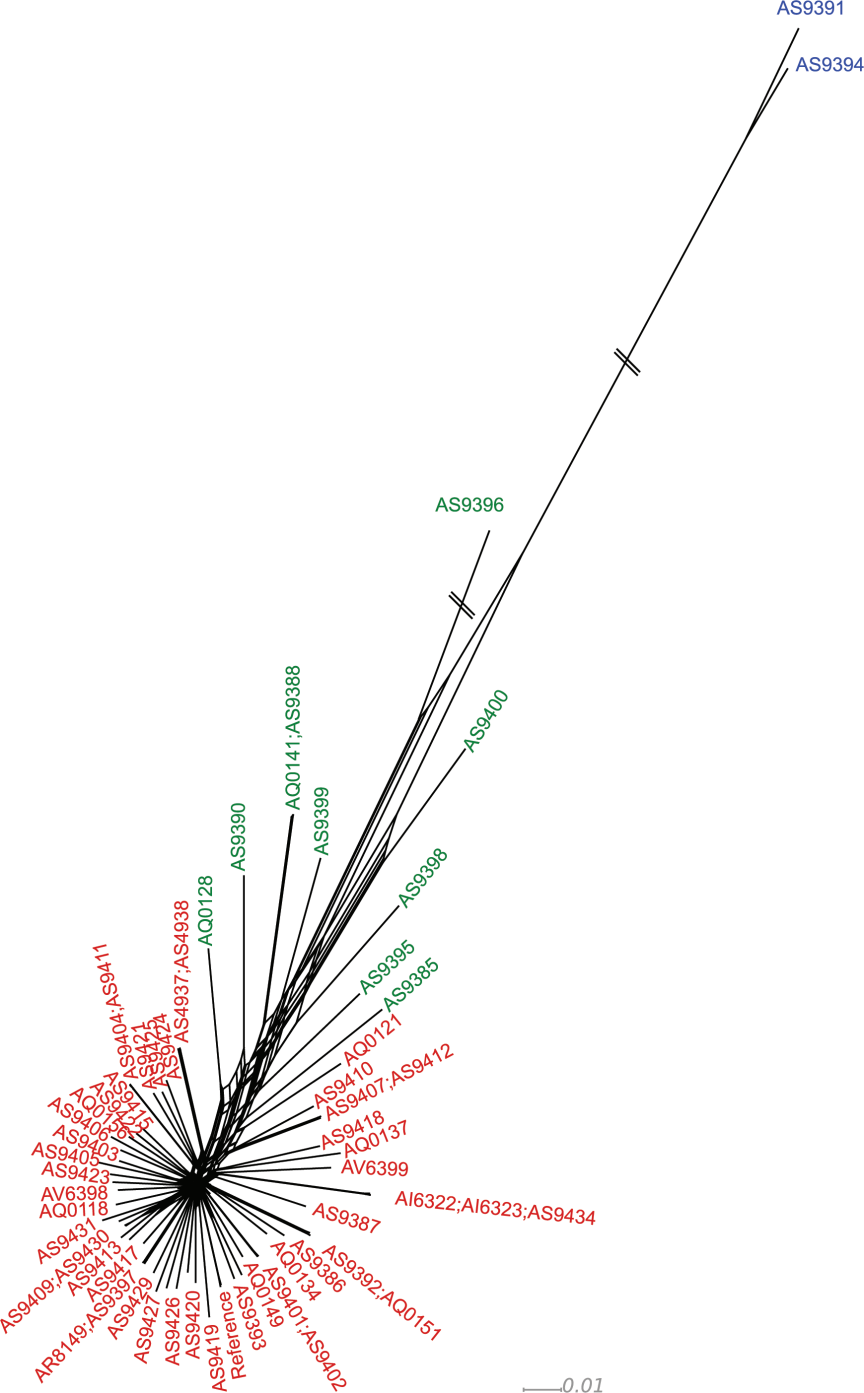
Phylogenetic network of sequenced *L. acicola* isolates. Isolates colored in blue represent those belonging to the NL, those in red belong to the SL, and those in green contain introgression from the NL to the SL based on Structure.

Given the results found with marker genes, we extended the search for introgression patterns to the entire genome. For this, we first calculated the number of SNPs per Kb with respect to the reference in the different isolates. Most strains had low SNP densities, ranging from 0.79 SNPs/Kb to 2.58 SNPs/Kb (see Table S2), indicating a recent shared ancestry with the reference. However, three isolates deviated from this pattern, showing a higher number of SNPs with respect to the reference. Two of these divergent isolates correspond to those assigned to the NL based on the marker gene analysis and had between 10.6 and 10.9 SNPs/Kb. The third divergent isolate (AS9396), which was assigned to the SL based on marker genes, showed an intermediate SNP density (5,06 SNPs/Kb). We plotted the SNPs/Kb for genomic windows of 5 Kb and observed that most SL strains presented a bimodal distribution with a large peak at 0 SNPs/Kb and a second, smaller peak at 2.5 SNPs/Kb, which is similar to the peak observed in NL strains and could indicate regions of introgression from the NL (Fig. 3A). These results are congruent with the evolutionary distances observed in a phylogenetic tree reconstructed from SNP data (Fig. 3B). And are also verified by the reconstruction of a network tree (see Materials and Methods) which shows clear signs of reticulation affecting not only strain AS9396 but also additional strains (Fig. 4).

To explore which strains were affected by introgressions, we calculated the genetic structure of the population using Structure (20). This analysis supported the presence of two main ancestral genetic populations, consistent with the previously proposed NL and SL (see Fig. 3C). As expected by the bimodal SNP distribution found in other strains, a set of nine additional isolates were inferred by Structure as having signs of admixture between the two populations, always being the fraction belonging to the SL more prominent (see Table S2). Isolate AS9396, which showed an intermediate SNP density towards the reference, is the most admixed, showing almost 40% introgression from the NL ancestry.

Note though that none of the isolates that had introgressed marker genes were detected by Structure as having a mixed ancestry, nor did they stand out in the phylogenetic tree or the network. This indicates, again, that marker genes are not good proxies for genome-wide patterns but also that Structure has a limited resolution and that additional strains may have lower levels of introgression that are not detected by Structure.

**Fig 5:**
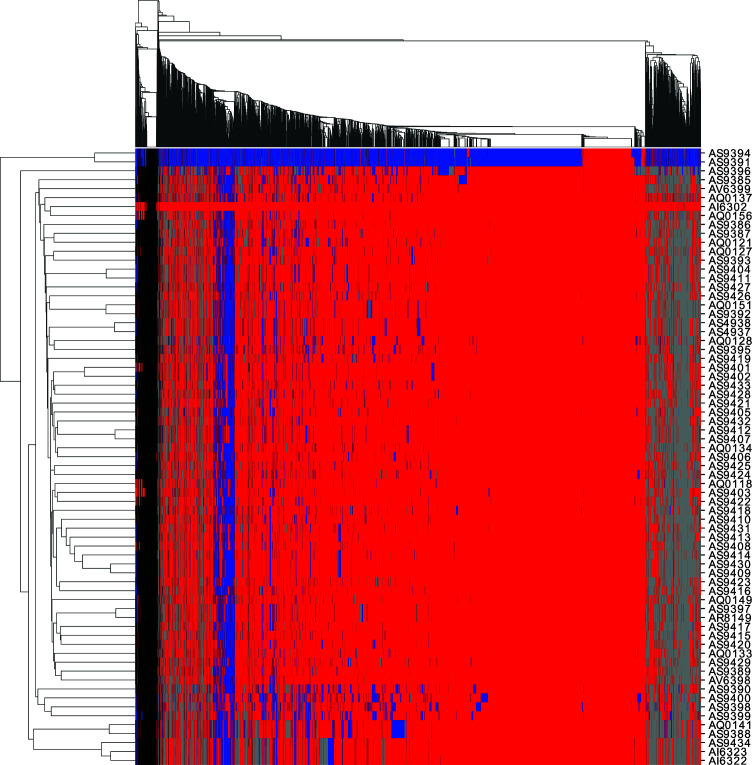
Heatmap showing genomic windows assigned to the SL (red), NL (blue), undetermined (gray), or unmapped (black) on a similarity threshold of 2 SNPs/Kb to the reference. Columns indicate 5 Kb windows and are clustered together according to the pattern of ancestry observed in the different isolates. Rows indicate each of the isolates sequenced in this study.

To test this hypothesis and investigate introgressed regions, we split the reference genome into non-overlapping 5 Kb windows and assigned each window to either the same population as the reference genome which belongs to the SL (less than 2 SNPs/Kb) or the NL (more than 2 SNPs/Kb and more than half of the SNPs predicted in the window match the SNPs found in the two Northern strains) (see Fig. 5). Windows that do not fulfill either criterion were considered undetermined. The first consideration is that, when comparing the NL strains to the SL reference, only 13.8% of the windows analyzed were below the 2 SNPs/Kb threshold, indicating this threshold is appropriate to distinguish the two lineages. On the opposite end, the SL isolate AI6302 was the most similar to the reference, having 97.8% of the genomic windows with less than 2 SNPs/Kb. The remaining SL isolates had 64% to 84% of the windows under the 2 SNPs/Kb threshold. Importantly, all these strains showed at least a small percentage (3%–15%) of the windows classified as NL.

These results confirm patterns that are clearly indicative of admixture, which suggests that strains from the NL and SL not only co-occur but also that they are actively exchanging genetic material. To our knowledge, this is the first time that evidence for genetic introgression between these two lineages have been reported at a genome-wide scale. As only 3% of the sampled isolates belonged to the NL it would be expected that, following mating between SL and NL, the descendants are more likely to back-cross with members of the SL, accounting for the residual and largely diversified presence of the NL in the genomes of most strains.

### The genomic aftermath of introgression

*L. acicola* is a heterothallic species requiring two individuals of opposite mating types to reproduce sexually (MAT 1-1 and MAT 1-2). The presence of introgressed regions in all strains belonging to the SL points to the existence of mating between the two different *L. acicola* lineages in the studied region. How frequent such events are is unknown. We searched for the presence of the different MAT loci in the sequenced genomes. Out of the 69 isolates, 30 were MAT 1-1, including the reference, and the remaining 39, including the two NL isolates, were MAT 1-2 (see Table S2). To assess the variability of MAT 1-2, we took the AS9391 NL strain as a reference and assembled the reads into a *de-novo* Illumina assembly. Then we mapped the reads of all the other strains against this NL reference genome. We observed that the MAT 1-2 loci between NL and SL differed in four SNPs. We then scanned all the strains that had MAT 1-2 loci to see which origin they had. Interestingly, the 10 strains detected by structure as introgressed were MAT 1-2 and nine out of the ten had a mating loci corresponding to the NL. Most of the remaining MAT 1-2 strains were SL, except two that had the NL haplotype. Strains with MAT 1-1 loci on the other hand were more homogeneous, with only isolate AS9410 having three SNPs when compared to the reference. As we do not have a representative of the NL with a MAT 1-1 loci, it is unknown whether the SNPs present in this strain could originate from an introgression between SL MAT 1-2 and NL MAT 1-1 strains.

**Fig 6:**
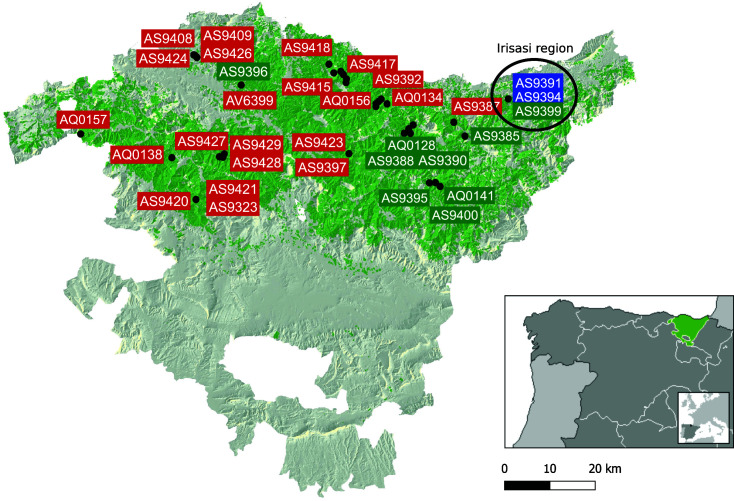
Geographic distribution of sampling sites for some of the strains used in this study. Blue, red, and green rectangles mark isolation sites for NL, SL, or introgressed (according to Structure) strains, respectively. The background area covered in green tones corresponds to the area of distribution of *Pinus radiata* arboretum in the Basque Country.

We then related the percentage of genomic windows belonging to the NL with the MAT loci in each strain and noticed that MAT 1-2 strains had a significantly larger percentage of NL windows (see Fig. S2). Given the small sampling of the NL in this study, it is difficult to extract conclusions but a plausible hypothesis is that the NL population in the Basque Country is expanding clonally with most crossings occurring between SL MAT 1-1 and NL MAT 1-2 strains. Alternatively, mating events may be rare and only a few such events happened, which were followed by clonal reproduction. In such a case, different numbers of mating events with SL strains after the first NL × SL cross would explain different levels of introgression across strains. In support of this last hypothesis is the geographic distribution of strains with larger introgressed regions seen in Fig. 6. The two NL strains were found in the location of the Irisasi arboretum [in the arboretum AR22 located in Irisasi (Gipuzkoa) (https://reinfforce.iefc.net/es/arboreta/ar22/)] and most of the introgressed strains are isolated in or close to that region. The only exception was strain AS9396 which may have reproduced clonally to maintain the high level of introgression or may have emerged from a different mating event. Still, we found that introgressed strains share, on average, 45% of NL windows, suggesting either a common origin or a convergent tendency to keep the same introgressed regions.

Beyond the MAT locus, other signs of introgression are found across the genome. We searched for genes that showed signs of introgression, having an SNP pattern that matched the one in the two NL strains. Across all strains, we found 3,237 genes that had been introgressed in at least one of the strains which accounts for one-third of the total coding genome. In all, 73 of those genes were introgressed in at least 50% of the strains included in this study. We searched whether any COG or GO terms were enriched in any of these sets, but no enrichment was found.

We then used read mapping to search for genes missing in the two NL strains. In all, 63 genes were identified as missing because more than 75% of their coding sequence was not mapped by reads. Among those, eight were exclusively missing in the two NL strains. While most of the proteins encoded by these genes were of unknown function, one of the proteins was annotated as an Aspartyl protease (LECACI7A000754P1). Although still poorly studied, aspartyl proteases have been associated with plant cell wall degradation in other plant pathogenic fungi (21). However, this particular protease is not predicted to be secreted and therefore is unlikely to act in such a role. Among genes lost in additional strains, there is a UDP-glucose/GDP-mannose dehydrogenase (LECACI7A003137P1) which could be related to the degradation of nucleotide-linked sugars which can be part of plants’ hemicellulose.

### Phenotypic variability

**Fig 7:**
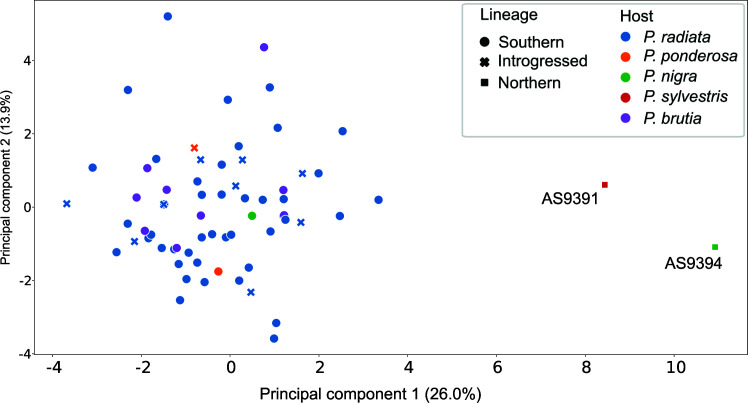
PCA based on enzymatic activity of 20 enzymes. Markers are based on the lineage: circles for the SL, squares for the NL, and crosses for the strains that are introgressed according to Structure. Colors relate to the host pine species.

Little is known about the phenotypic differences that exist between the NL and SL. Huang et al. (14) reported different responses to temperature between members of the two lineages which would account for their adaptation to different environments. Here, we show that SL and NL strains can be found in the same area, indicating that these differences do not totally exclude geographical overlap. To gain insight into other functional differences between these two lineages, we analyzed the enzymatic activity on 20 different compounds for 66 of the strains, scoring the activity levels from one (low activity) to five (high activity) (see Fig. S3; Table S3). A principal component analysis shows a clear difference between the two NL strains and all the other strains, including those of mixed ancestry as derived from Structure (Fig. 7; Fig. S4). We tried to relate the results with other factors, such as host, mating type, and localization. Based on an Adonis test, we found a significant association between phenotype and the host source but not with either the mating type or the sampling location. Interestingly, all strains that were detected as having introgression with the NL by Structure did not show any phenotypic difference to all the other strains of the SL, suggesting introgressed regions do not drive these phenotypes.

We assessed the enzymatic activities that differed the most among strains from the SL, including strains with introgressed regions, and strains from the NL (see Fig. S5). Two activities that were low in strains from the NL but much higher in the SL are alpha and beta-galactosidases which have been associated with the degradation of the plant cell wall and are considered a virulence factor (22, 23). Alpha-galactosidases are involved in the degradation of O-acetylgalacto-glucomannan, specifically in the hydrolysis of terminal α-1,6-linked galactose residues (24, 25). O-acetylgalacto-glucomannans are important hemicellulosic components of softwoods, comprising up to 25% of their dry mass. β-Galactosidases are involved in the degradation of Xyloglucan, another hemicellulose found in the primary cell wall of the majority of higher plants (26, 27). NL strains also had no discernible activity of N-acetyl-ß-glucosaminidase, which has been associated with chitin degradation, a fundamental structural component of the cell wall of fungi. In other fungi, N-acetyl-ß-glucosaminidase activity was induced during antagonistic fungal interactions and its role was related to the degradation of chitobiose generated during cell wall decomposition (28, 29). Strains from the SL have been reported to be more virulent than the NL (30). This could be partially attributed to the lower activity of some enzymes such as the beta-galactosidases shown here. In addition, the higher activity of N-acetyl-ß-glucosaminidase in the SL isolates could be translated into a higher competition capacity of this lineage in natural conditions when competing with other fungi than the NL *L. acicola*. Also, it could be a factor explaining the predominance of the SL in the area of study.

### Conclusion

We present here the first genome assembly of the plant pathogenic fungi *L. acicola*, providing an important resource to support research and measures to control this expanding pathogen that represents a growing threat to pine tree forests. The comparison of the genome of *L. acicola* to other, closely related Dothideomycetes species showed a high similarity in terms of the number of secreted and CAZY proteins, with no distinct patterns shown by the three pine pathogens included in the set. Interestingly, we found a conserved secondary metabolism gene cluster between *L. acicola* and another pine pathogenic fungi *D. septosporum,* although it remains unclear whether this plays a role in pine colonization or infection. The sequencing of an additional set of 69 strains from forests in the north of Spain where this pathogen has expanded in recent years revealed the presence of two NL strains, which are uncommon in southern Europe. Analysis based on Structure and SNP comparison showed that recombination is prevalent among strains from the two main *Lecanosticta* lineages and that admixture has probably been ongoing for a long time given the different percentages of recombined regions in the different strains. Admixture between NL and SL is a finding without precedents worldwide that increases the previously expected evolutionary potential, production of genetic variation and the ability to adapt to new conditions of L. acicola, which could represent a threat to the already established management measures to control brown spot needle blight. The experimental measurement of a set of enzymatic activities in the different strains of *L. acicola* showed a clear divide between the phenotypes of the NL and SL and pointed to a reduced activity of alpha and beta-galactosidases in the NL species. This phenotypic difference could explain the observation that strains from the NL tend to be less virulent than members of the SL.

## MATERIALS AND METHODS

### Strains collection and DNA extraction

The collection of isolates comes from a previous study of the population described in Mesanza et al, 2023 (submitted) (see Table S2). For *L. acicola* gDNA extractions, 0.25 cm^2^ of mycelia were cut, grinded, and grown in 50 mL yeast extract peptone dextrose broth in 250 mL flasks at 22°C and 180 rpm. After a week, the contents of the flasks were poured in 50 mL Falcons and centrifuged for 10 minutes at 5,000 rpm (2688 g), supernatants were discarded and mycelia were frozen at −80°C for at least 2–3 hours. Samples were lyophilized overnight, and 200 mg of dry material was used for the extraction with the Quick-DNA Fungal/Bacterial Miniprep Kit (Zymo Research, California) following the manufacturer’s instructions.

### Long-read whole-genome sequencing

Genomic DNA of *Lecanosticta acicola* was quality controlled using 1% agarose gel electrophoresis and Qubit dsDNA HS Assay kit (Thermo Fisher Scientific), then it was re-purified using AMPure XP Beads (Agencourt, Beckman Coulter) adding 0.4 vol (Vol/Vol) to the sample. The rapid sequencing gDNA-low input PCR barcoding (SQK-PBK004) protocol (Oxford Nanopore Technologies, ONT) was used to prepare the sequencing library starting with 0.17 micrograms of restricted integrity gDNA without a fragmentation step. The DNA was repaired using the NEBNext FFPE Repair Mix (New England Biolabs, NEB), end-repaired and adenylated with the NEBNext Ultra II End Repair and A-Tailing Module (NEB) and then adapters which contain primer binding sites are ligated onto the prepared ends. The kit contains 12× primer pairs which can be used to amplify each sample, for the unique sample used barcode primer BP03 which contains a barcode and 5′ tags which facilitate the ligase-free attachment of Rapid Sequencing Adapters (RAP) added after the PCR amplification (15 cycles) using LongAmp Hot Start Taq 2X Master Mix (NEB).

The sequencing run was performed on a GridIon instrument (ONT) using the FLO-MIN106D flow cell (ONT), according to the manufacturer’s recommendations. In brief, first, the MinKNOW interface QC (ONT) was run to assess the flow cell quality followed by flowcell priming. The sequencing library was mixed with a running buffer, Library Loading Beads (Oxford Nanopore Technologies), and nuclease-free water and loaded onto the “spot on” port for sequencing. The sequencing data were collected for 48 hours. The quality parameters of the sequencing runs were further monitored by the MinKNOW platform.

### Short-read whole-genome sequencing

The short-insert paired-end libraries for the whole-genome sequencing were prepared with PCR-free protocol using KAPA HyperPrep kit (Roche). The libraries were quality controlled on an Agilent 2100 Bioanalyzer with the DNA 7500 assay (Agilent) for size and quantified by Kapa Library Quantification Kit for Illumina platforms (Roche).

The libraries were sequenced on NovaSeq6000 (Illumina) or HiSeq 4000 (Illumina) in paired-end mode with a read length of 2 × 151 bp following the manufacturer’s protocol for dual indexing. Image analysis, base calling, and quality scoring of the run were processed using the manufacturer’s software Real Time Analysis (RTA 3.4.4, resp. RTA 2.7.7) and followed by the generation of FASTQ sequence files.

### Reference genome assembly

DNA sequencing reads were pre-processed as follows. The paired-end short reads were adaptor-trimmed with cutadapt (31) v1.2.1 without quality trimming (-q = 0). The trimmed reads were downsampled to keep 4,990,205 read pairs, accounting for 40×. Nanopore reads sequenced on the ONT GridION platform were filtered to retain reads with a minimum mean base quality of 7 and minimum length of 4.5 kb, resulting in 811,999 reads, accounting for 110× coverage (Table S4).

The processed Illumina and ONT sequences were assembled with Unicycler v0.4.6 (32), using the default normal mode. We screened the genome assembly to detect contaminants at the species level using BlobTools v1.1 (parameters: e-value 10e-25 -max_target_seqs 25 -culling_limit 2 against the NCBI nucleotide collection (nt database updated on 30 Dec 2019) (33) (Fig. S6). There was strong evidence for contamination with non-fungal species. Two contigs matching Pseudomonas oryzihabitants, including its complete genome, and 1,001 contigs matching Gadus morhua were removed. The final assembly had a genome size of 30.4 Mb found in 329 contigs with an N50 of 290,931 bp. Genome completeness was assessed using BUSCO v5.4.7 (34, 35) with the fungi_odb10 database (Table S5).

### Genome annotation

Repeats present in the *Lecanostica acicola* genome assembly were annotated with RepeatMasker v4-1-2 (http://www.repeatmasker.org) using the custom repeat library available for fungi. Moreover, a new repeat library specific to our assembly was made with RepeatModeler v1.0.11. After excluding repeats belonging to repetitive protein families (performing a BLAST (36 search against UniProt) from the resulting library, Repeat Masker was run again with this new library to annotate the specific repeats.

The gene annotation was obtained by combining protein alignments and *ab initio* gene predictions. First, the complete *Dothistroma septosporum* proteome was downloaded from UniProt in March 2020 and aligned to the genome using Spaln v2.4.03 (37). *Ab initio* gene predictions were performed on the repeat masked assembly with four different programs: GeneID v1.4 (38), Augustus v3.3.4 (39), Genemark-ES v2.3e (40), and GlimmerHMM (41). Genemark-ES, which runs in a self-trained manner, was executed in “fungus” mode. The other programs were trained using the protein alignments of *Dothistroma septosporum*. Training parameters for Augustus can be found at (https://github.com/ERGA-consortium/pipelines/tree/main/annotation/resources/AUGUSTUS/Lecanosticta_acicola) and for geneID they can be found at (https://github.com/guigolab/geneid-parameter-files). Finally, all the data were combined into consensus CDS models using EvidenceModeler-1.1.1 (EVM) (42). Functional annotation was performed with Blast2go (43) and Interproscan 5 (44) Annotations were combined which produced the final functional annotation results.

### Phylome reconstruction

A phylome, the collection of phylogenetic trees for each gene encoded in a genome, was reconstructed for the genome of *L. acicola*. We selected a set of 28 Mycosphaerellales genomes from NCBI (see Table S1). Those that did not contain an annotation in NCBI were annotated with Augustus (45). The phylome was reconstructed using the phylomeDB pipeline (15). First, starting from each protein of *L. acicola* a BlastP (36), a search was performed against the database of proteomes reconstructed for this project. Blast results were filtered based on e-value (< 1e-05) and overlap (50%) and the best 200 hits were kept. The selected sequences were then aligned using three different programs: MUSCLE v3.8.1551 (46), MAFFT v7.407 (47), and KALIGN v2.04 (48). Sequences were aligned twice, first in forward and in reverse. M-coffee v12.00 (49) was then used to obtain a consensus alignment based on the six pre-calculated alignments. This alignment was then filtered using trimAl v1.4.rev15 (50) (parameters: -ct 0.1666666, -gt 0.1 and -cons 30). The final alignment was then used to reconstruct a maximum phylogenetic tree using IQTREE v1.6 (51). odelFinder as implemented in IQTREE was limited to five models (DCmut, JTTDCMut, LG, WAG, VT). The number of free rate categories was limited to between 4 and 10. Support was calculated using 1,000 rapid bootstraps. In all, 9,561 trees were calculated and uploaded to phylomedb (15) with a PhylomeID 117.

EvolClust v1.0 was run on the species included in the phylome to search for conserved gene clusters among the species (17). EvolClust first calculates the background gene order conservation between pairs of species, then it obtains sets of gene clusters that are more conserved than the background and then groups them into families.

### Functional analysis of the reference genome

We ran signalP v5.0 (52) on all the proteomes included in the phylome. We then ran TMHMM on the same data set. We then filtered the results to keep only those proteins that had a probability to be secreted above 0.75 (probability of OTHER <0.25) and did not contain transmembrane domains (PredHel = 0 or PredHel = 1 if the predicted tmhmm falls within the first 60AA) (see Table S1).

We used the dbcan metaserver (version May 2022) to predict CAZY proteins using the three methods proposed. Predicted proteins were filtered by keeping only those that were predicted by at least two of the programs. The annotation of the families was taken preferentially from the HMM prediction as recommended (see Table S1). Based on the predicted families, we build a PCA to compare species based on their PCA content.

### Read mapping and SNP calling

For each of the 69 sequenced strains and the strain downloaded from NCBI, we used PerSVade v1.2.04 (18) to map reads to the reference genome and call SNPs. PerSVade obtains SNP predictions by correcting reads using Trimmomatic, aligning them to the reference genome using BWA, and then using three different SNP callers to predict SNPs (FreeBayes, BCFtools, and GATK Haplotype Caller). Minimum read coverage is set to 20 and haploid mode is used. Only SNPs that were called by at least two of the callers were considered (2xPASS). Read coverage for each position was calculated using bedtools v2.25 (53).

### Analysis of marker genes

Marker genes were downloaded from NCBI based on codes found in Table 1 of the publication by van der Nest and colleagues (12). For each marker gene and microsatellite region, a blastn search was performed against the reference genome. The boundaries for each sequence were obtained from the blast and the reference sequences were obtained. Then, the SNP calling done by PerSVade was scanned for the presence of SNPs in the corresponding regions, and for each strain, the SNPs were introduced in the reference sequence obtaining pseudo-sequences. The sequences were then aligned using mafft v7.508 (47) (parameters: default) and trimmed using trimAl v1.4.rev15 (50) (parameters -nogaps). IQTREE v2.2.0.3 (51) was then used to build a maker-based tree using default parameters and an ultra-bootstrap of 1,000. The tree image was built using the ETE v3 library (54).

### Strain tree reconstruction

A pseudo-alignment was reconstructed by substituting all positions in the reference genome that had an SNP in a given strain by the SNP. Regions that were poorly covered (coverage <20 reads) or that contained indels in any of the strains were also omitted. Positions that did not contain a SNP were also removed from the alignment. This resulted in a pseudo-alignment of 328,121 positions. IQTREE was then used to calculate the species tree. The best model according to the BIC criterion was TVM + F + ASC + R5. 1,000 rapid bootstraps were calculated. This alignment was also used to reconstruct a network phylogeny using SplitsTree v4.19.0 (55).

### Population analysis

The structure was used to study the population structure of the strains. First, 10 sets of 20,000 positions were randomly selected from the concatenated alignment constructed for the species tree reconstruction. The allele frequency was first established using two of the partitions. This was done by running Structure for each of the sets with a K = 1. Three independent runs were done per data set with a burnin of 10,000 and a run length of 20,000. In the three cases, the resulting allele frequency (lambda) was detected to be 0.36. With the allele frequency fixed to that value, we ran each of the 10 partitions with 9 different K values. In this case, each run was executed with a burnin of 20,000, and the MCMC was allowed to run for 100,000 generations. Then we used Structure Harvester (56) to establish the best K (see Fig. S7).

We then split the reference genome in non-overlapping windows of 5,000 bp. Only contigs with more than 100 Kb were considered for this analysis. First, we mapped the SNPs/Kb for each window and calculated the frequency for each strain (Fig. 3A). Then, for each window, the list of SNPs found in the two strains from the NL was taken. For each strain, the set of SNPs found in each window was compared to the consensus SNP set of the NL, and the number of common SNPs was counted. If the total number of SNPs was equal to or below 10, the window was assigned to the SL. If more than 10 SNPs were detected and more than 50% of SNPs in the window were the same as the ones found in the NL, then the genome fragment was considered to have been the result of introgression and originated in the NL. If the SNPs did not match the ones found in the NL, then this window was considered undetermined. Windows with no read mapping or that did not fulfill any of the previous conditions were tagged as either unmapped or undetermined. A clustered heatmap was built based on the inferred lineage of each window using seaborn (57).

Genes were checked to see whether they had the same SNP pattern found in the strains of the NL. Genes with the same pattern were considered to be introgressed.

We then checked which genes from the SL were missing in the NL by relating the read coverage to gene positions. Genes with less than 25% of their gene covered were considered lost.

### Enzymatic profiling

A broad enzymatic profile of 70 isolates was determined using an API ZYM system (BioMérieux, Marcy l’Etoile, France). API ZYM is a semi-quantitative micromethod designed for the research of enzymatic activities. The technique applys to all specimens (microorganisms, cell suspensions, tissues, biological fluids, etc.). It allows the systematic and rapid study of 19 enzymatic reactions using very small sample quantities. The system consists of a strip with 20 microwells (cupules), the base of which contains the enzymatic substrate and its buffer. This base allows contact between the enzyme and the generally insoluble substrate. Inoculum was obtained by plating spore suspensions of each isolate in pine minimum medium with glucose (58, 59). After 1 week, 20 colonies per isolate were selected and inoculated in each well of the API ZYM strip, the strips were incubated at room temperature for 7 days. Enzymatic activity was graded from 0 to 5, being 0 no activity, 1 low activity, 2–3 moderate activity, and 4–5 high activity (60) (see Table S3).

A PCA was built based on the enzymatic activities using the Scikit learn v1.2.1 Python Library. We then assessed associations between four different variables (lineage, mating loci, host, and sampling location) and the overall enzymatic activity of the different strains by performing permutational multivariate analysis of variance (PERMANOVA) using the adonis function from the Vegan R package (v. 2.5–6) using the Bray-Curtis dissimilarity distance.

## Data Availability

All sequencing data has been deposited at ENA (PRJEB62799)

## SUPPLEMENTAL MATERIAL

Supplemental material msystems.00928-23-s0001.pdf Supplemental table captions and Figures S1 to S7.

Supplemental tables msystems.00928-23-s0002.xlsx Tables S1 to S5.

ASM does not own the copyrights to Supplemental Material that may be linked to, or accessed through, an article. The authors have granted ASM a non-exclusive, world-wide license to publish the Supplemental Material files. Please contact the corresponding author directly for reuse.
