# Supplementary material for: Genomics of the expanding pine pathogen Lecanosticta acicola reveals patterns of ongoing genetic admixture
Source: mSystems. 2024 Feb 16;9(3):e00928-23. doi: 10.1128/msystems.00928-23 (PMC10949461; doi:10.1128/msystems.00928-23)
Supplement: Supplemental material — Supplemental table captions and Figures S1 to S7. [file msystems.00928-23-s0001.pdf]

Supplementary material for: Genomics of the expanding pine pathogen Lecanosticta acicola reveals patterns of ongoing genetic admixture.

Supplementary Table legends

Table S1: List of strains included in the study. Table includes strain codes, host species, sampling localization, predicted number of SNPs when compared to the reference, SNPs / kb, fraction of the genome allocated to the SL and the NL as predicted by structure and the MAT loci.

Table S2: List of species included in the phylome and functional characterization of L. acicola. Table includes the taxonomic ID of the species, the species name, the size of the proteome, and the number of secreted and CAZy proteins predicted and the percentages they represent.

Table S3: Enzymatic activities detected for the analyzed strains. Columns indicate each of the 20 enzymes considered.

Table S4: Estimated coverage of the different sequencing libraries. Read length information for Oxford nanopore corresponds to the N50 read length. Sequencing coverage was obtained using the assembly span 30.4Mb.

Table S5: Statistics of the genome assembly of *Lecanosticta acicola*

Supplementary Figures and Legends

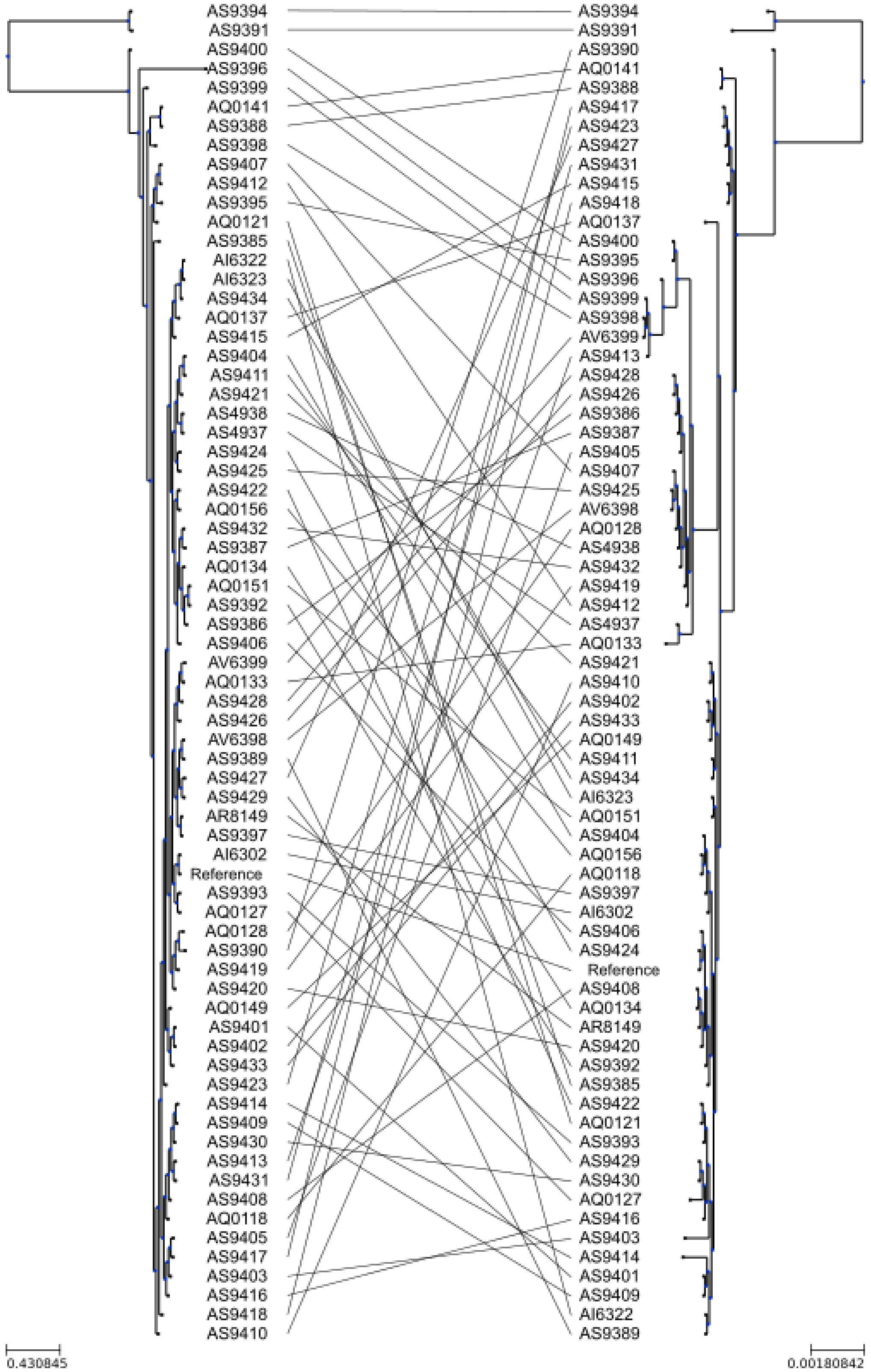

Fig. S1: comparison between the tree built from the concatenation of SNPs across the whole genome (left) and the tree based on microsatellite sequences (right). Lines link the same strain in the two trees.

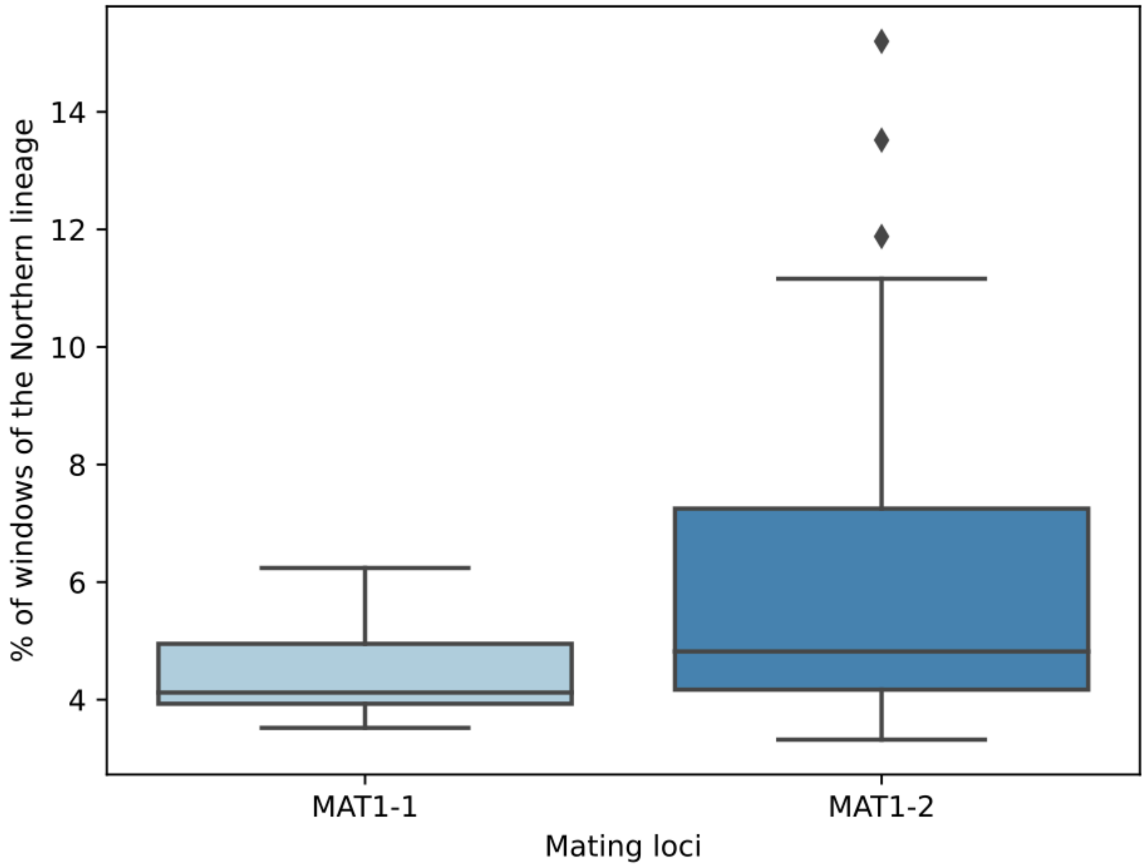

Fig. S2: Boxplot representing the percentage of windows from the NL strains introgressed in the strains with a MAT 1-1 and a MAT 1-2 loci. The two distributions are significantly different (p-value 0.00345).

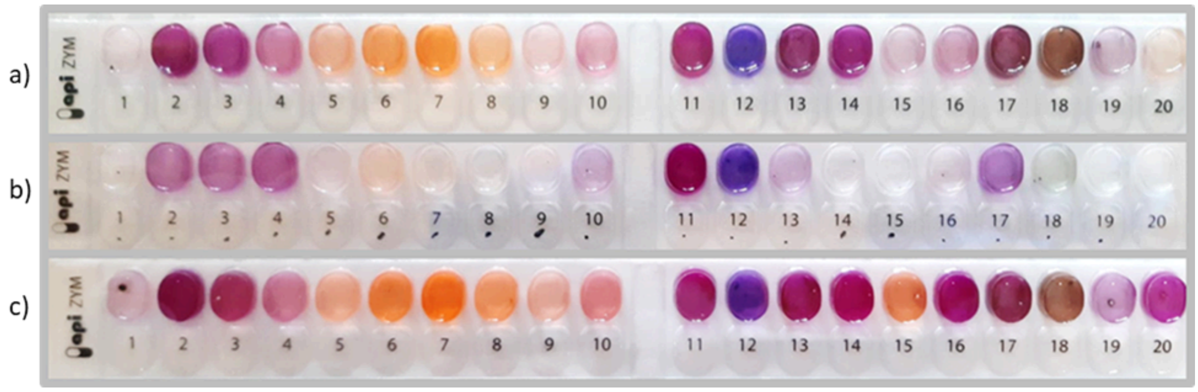

Fig. S3: Example of phenotypic testing, showing different enzymatic profiles for different *L. acicola* isolates a) AQ0156 b) AS9394 and c) AQ0121.

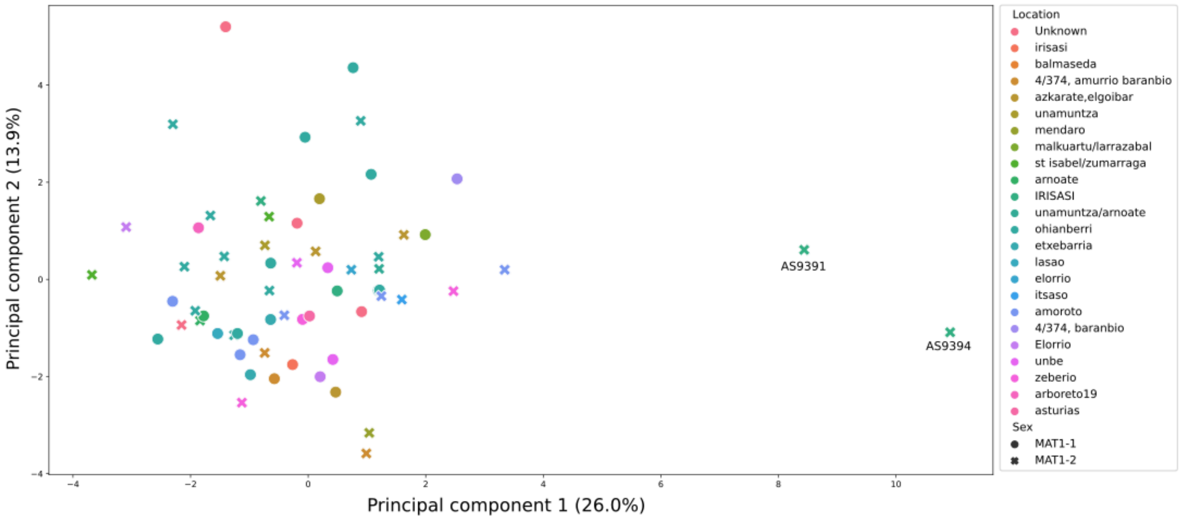

Fig. S4: PCA based on enzymatic activity of 20 enzymes. Markers are based on the MAT loci: circles for MAT 1-1 and crosses for MAT 1-2. Colours relate to the location where the strain was sampled from.

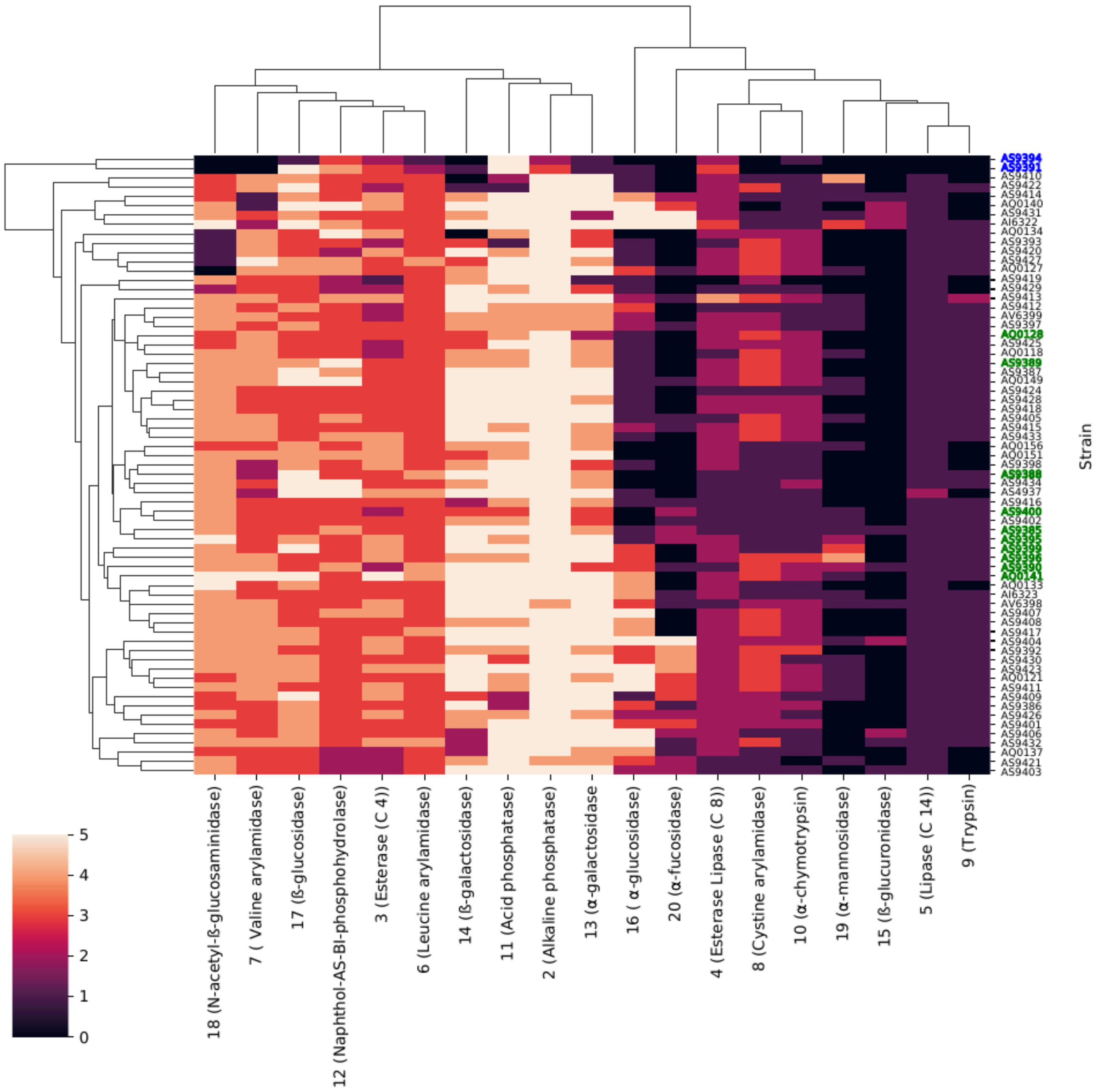

Fig. S5: Heatmap depicting the enzymatic activity produced by each strain, ranked from 0 (minimum) to 5 (maximum). Strain names coloured in blue indicate the two strains from the Northern lineage. Strain names in green indicate strains with introgressions as detected by Structure.

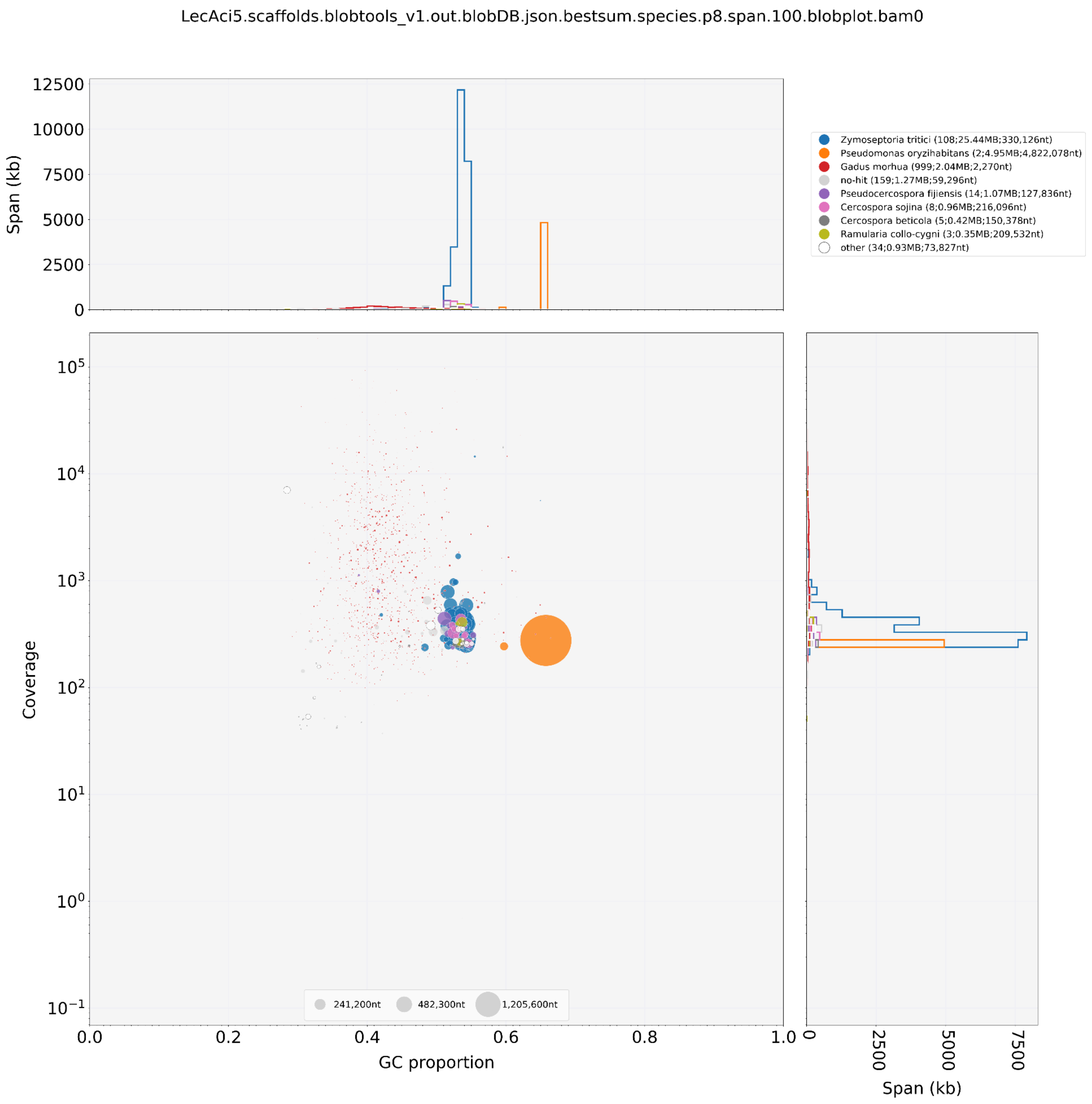

Fig. S6. Blobplot showing draft assembly sequences plotted by GC content (x-axis), Illumina coverage (y-axis), sequence length (blob size) and taxonomic assignment by colour according to the legend.

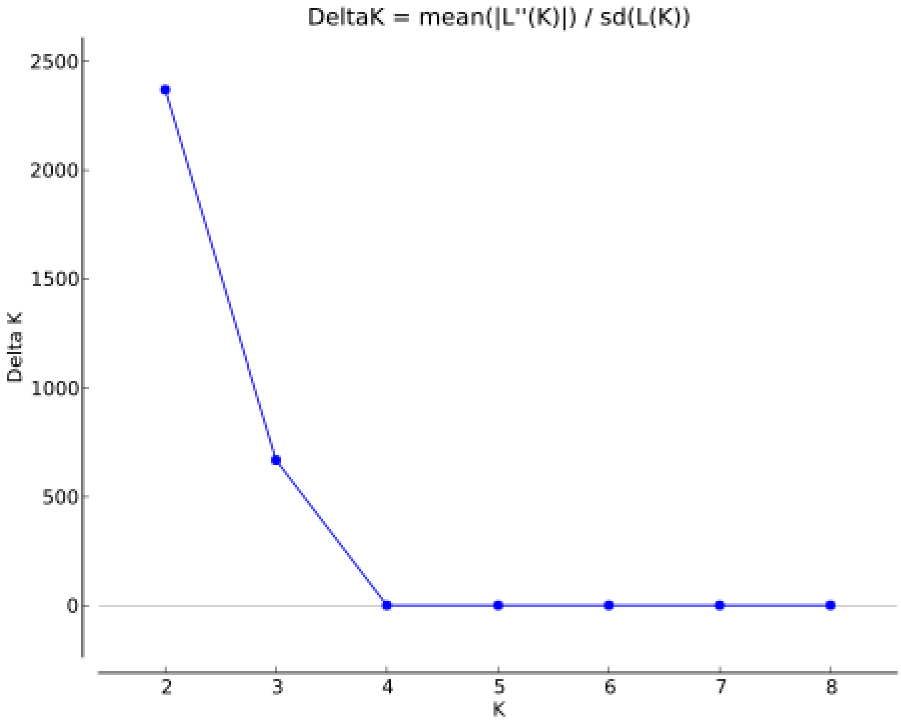

Fig. S7: Graph depicting the delta K as predicted by Structure Harvester.
